# Case Report: Dual-chamber pacemaker for hypertrophic cardiomyopathy with bradyarrhythmia and idiopathic pericardial effusion: a report of two cases and literature review

**DOI:** 10.3389/fcvm.2025.1518000

**Published:** 2025-02-27

**Authors:** Chang Liu, Fei Zheng, Yuxia Gao, Zheming Wang, Xinyu Zhang, Xiuqing Tian

**Affiliations:** ^1^ Department of Cardiology, Shandong Provincial Medical and Health Laboratory of Cardiac Electrophysiology and Arrhythmia, The First Affiliated Hospital of Shandong First Medical University & Shandong Provincial Qianfoshan Hospital, Jinan, China; ^2^ Department of Cardiology, Jinan Changqing District Hospital of Traditional Chinese Medicine, Jinan, Shandong, China

**Keywords:** hypertrophic cardiomyopathy, sinus node dysfunction, dual-chamber pacemaker, pericardial effusion, case report

## Abstract

**Background:**

Hypertrophic cardiomyopathy (HCM) is an autosomal dominant disorder characterized by asymmetric hypertrophy of the ventricles and the ventricular septum, leading to subsequent left ventricular outflow tract (LVOT) obstruction and diastolic dysfunction. Typically, patients with HCM experience sinus tachycardia and sinus arrest relatively infrequently. In addition, the concurrent occurrence of HCM with non-surgically induced (ablation or myectomy) bradyarrhythmia and idiopathic pericardial effusion in adult patients has not been previously reported.

**Case summary:**

In this report, we present two elderly female patients with HCM who exhibited sinus bradycardia and sinus arrest, one of whom also presented with moderate pericardial effusion, they all presented with chest tightness. To manage the complex comorbidities, we opted for dual-chamber pacemaker implantation. Subsequent examinations and follow-up revealed that pacing significantly reduced LVOT obstruction and corrected heart rhythm. Additionally, there was no significant progression of pericardial effusion.

**Discussion:**

The primary strategies for alleviating LVOT obstruction involve altering the structure of the septum, including septal myectomy (SME), alcohol septal ablation (ASA), and septal radiofrequency ablation. Meanwhile, a dual-chamber pacemaker can treat HCM by changing the sequence of myocardial contraction. Although pacemakers have been considered an inferior alternative due to their relatively large residual obstruction, their benefits may be significantly underestimated. This report underscores the additional efficacy of dual-chamber pacemakers in managing HCM, particularly in patients complicated by sinus node dysfunction and idiopathic pericardial effusion.

## Introduction

Hypertrophic cardiomyopathy (HCM) is the most common hereditary cardiovascular disease which characterized by idiopathic, nondilated ventricular thickening. This structural abnormality leads to a significant increase in intraventricular pressure and oxygen demand, which can progressively impair the heart's diastolic and conductive functions (1). Patients with HCM face an elevated risk of life-threatening arrhythmias and sudden cardiac death (SCD). While ventricular fibrillation is the primary cause of sudden death in most HCM patients, it is important to note that sudden death can also result from sinus node dysfunction (SND), such as sinus bradycardia and sinus arrest. SND is a relatively uncommon complication associated with HCM, and its risk is heightened in the presence of myocardial ischemia or heart failure.

Based on the symptoms, clinical signs, and auxiliary examinations, our two elderly patients were diagnosed with HCM complicated by SND, with one patient also exhibiting moderate pericardial effusion. These patients faced significant risks of SCD, and managing their conditions posed considerable challenges. Acknowledgedly, dual-chamber pacing has not been the superior option in the invasive treatment of HCM. However, our postoperative follow-up revealed that the pacemaker not only alleviated the obstruction and maintained an appropriate sinus rhythm but also effectively controlled the pericardial effusion. Through these two case studies, we believe that the presence of potential benefits and various complications of dual-chamber pacing should be thoroughly evaluated in future HCM treatment strategies.

## Case presentation

### Case 1

An 84-year-old female, presenting with a one-month history of chest tightness and palpitations that had worsened over the past two days, was admitted to our ward on January 25, 2023. Her accompanying symptoms included a cough with sputum, sore throat, and drowsiness. She has a medical history of diabetes and hypertension, and underwent radical surgery for bladder malignancy ten years ago. Family history is unknown.

Cardiac examination revealed a positive finding of a grade 3/6 systolic murmur at the left sternal border in the 3rd and 4th intercostal spaces, with a heart rate of 40 beats per minute. Laboratory tests indicated poor heart and kidney function, with BNP levels at 2052.5 pg/ml, creatinine at 139.5 µmol/L, urea nitrogen at 15.49 mmol/L, and potassium at 6.9 mmol/L. Subsequent echocardiography revealed severe obstruction of the left ventricular outflow tract (LVOT) and increased blood flow velocity (maximum pressure gradient of 111 mmHg, maximum flow velocity of 528 cm/s, and mean pressure gradient of 51 mmHg), accompanied by significant systolic anterior motion (SAM) of the anterior mitral leaflet and basal septal hypertrophy (16 mm) protruding into the LVOT. Additionally, the Holter monitor detected bradycardia (heart rate less than 50 beats per minute), accounting for approximately 17.5% of the total heartbeat, with an average heart rate of 55 bpm, a minimum heart rate of 32 bpm, and four sinus arrests (RR > 2 s).

**Figure 1:**
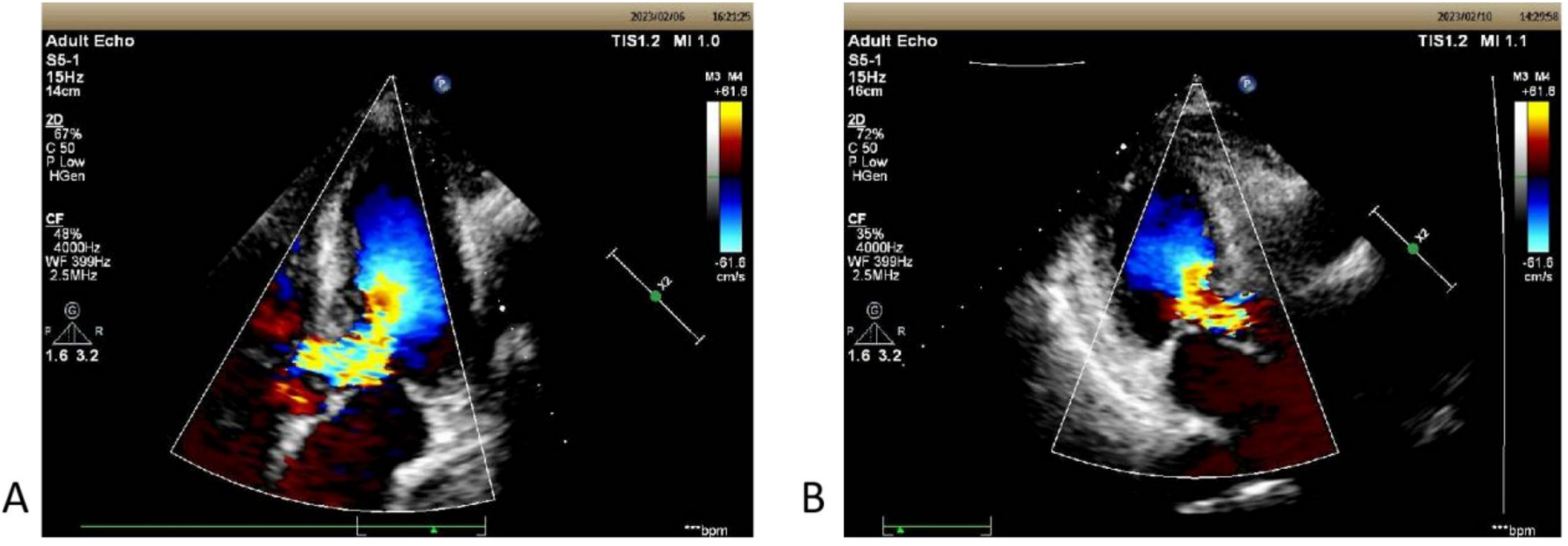
**(A)** The echocardiographic view of patient 1 before operation with septal hypertrophy measuring 1.60 cm. Severe left ventricular outflow tract obstruction with increased blood flow velocity in the left ventricular outflow tract at rest, maximum flow velocity of 528 cm/s, maximum pressure gradient of 111 mmHg, and mean pressure gradient of 51 mmHg. **(B)** On the third postoperative day, the second echocardiography showed: The left ventricular outflow tract velocity with a maximum flow velocity of 244 cm/s and a maximum pressure gradient of 24 mmHg.

**Figure 2:**
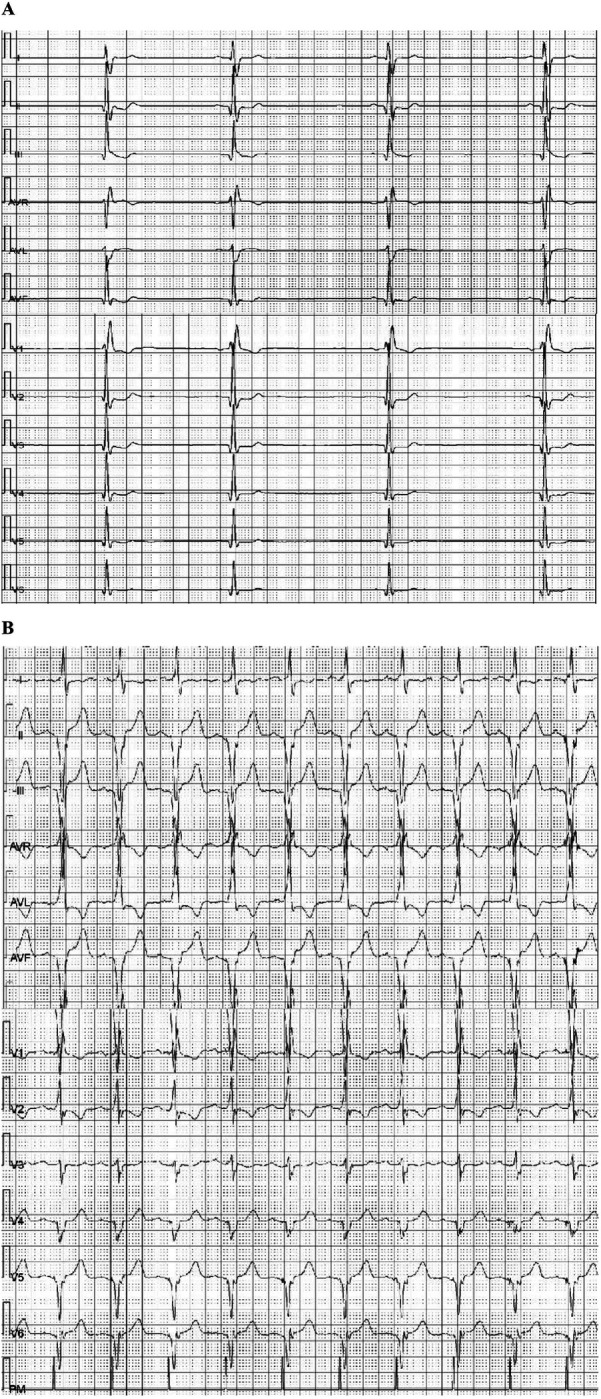
Holter examination of patient 1: **(A)** twelve-lead Holter ECG (before pacing) depicting sinus bradycardia (55 bpm), with the slowest heart rate of 32 beats per minute. **(B)** Holter ECG (day 3 after pacing) showed that pacing rate was 99.8%, with an average heart rate of 67 beats per minute, the slowest being 59 beats per minute, and the fastest 84 beats per minute.

Due to the complications of bradyarrhythmia and sinus arrests, the use of β-blockers or calcium channel antagonists for alleviating obstruction was relatively contraindicated. After comprehensive consideration, it was determined that dual-chamber pacemaker implantation could potentially alleviate the obstruction by altering the pacing sequence of the cardiac chambers and interventricular septum, as well as improve the ventricular rate. During the early stages of hospitalization, treatments including potassium reduction, glucose control, diuresis, and myocardial nutrition significantly improved the patient's general condition. With the consent of the patient and her family, a dual-chamber pacemaker was implanted on February 7, 2023. Under local anesthesia, atrial and ventricular electrodes were successfully fixed to the right atrial appendage and the right ventricular apex via the superior vena cava, operating in DDD mode. On third postoperative day, a repeat transthoracic echocardiogram revealed a significant decrease in the peak instantaneous gradient at the basal left ventricular segment from 111 to 24 mmHg, with the SAM of the mitral valve no longer present, and the maximum flow velocity of the LVOT reduced to 244 cm/s (Figure 1). The repeat holter indicated that pacemaker working well, pacing heart rate accounting for approximately 17.5% of the total heartbeats, with an average heart rate of 67 bpm, a minimum heart rate of 59 bpm, and no instances of sinus pause were observed (Figure 2). Additionally, the BNP level decreased to 48.59 pg/ml on the day before discharge. The patient continued taking metoprolol after discharge. Following a 2.5-month post-discharge follow-up, the patient's general condition remained stable, with a cardiac rate of 77 bpm and a maximum rate of 87 bpm. Her BNP level was 81.11 pg/ml, echocardiographic showed resting LVOT forward flow velocity: 244 cm/s.

### Case 2

A similar case involves an elderly female patient, aged 71, with a history of hypertension, who presented to our hospital on March 17, 2023. She had been experiencing dizziness and chest tightness for two months before admission, accompanied by nausea, tinnitus, and palpitations. In 2011, she was diagnosed with hypertrophic cardiomyopathy, and in 2019, she was diagnosed with sinus bradycardia. Since her diagnosis, she had been taking antihypertensive medications and Chinese herbal remedies. Her past treatment effects and family history is unknown.

**Figure 3:**
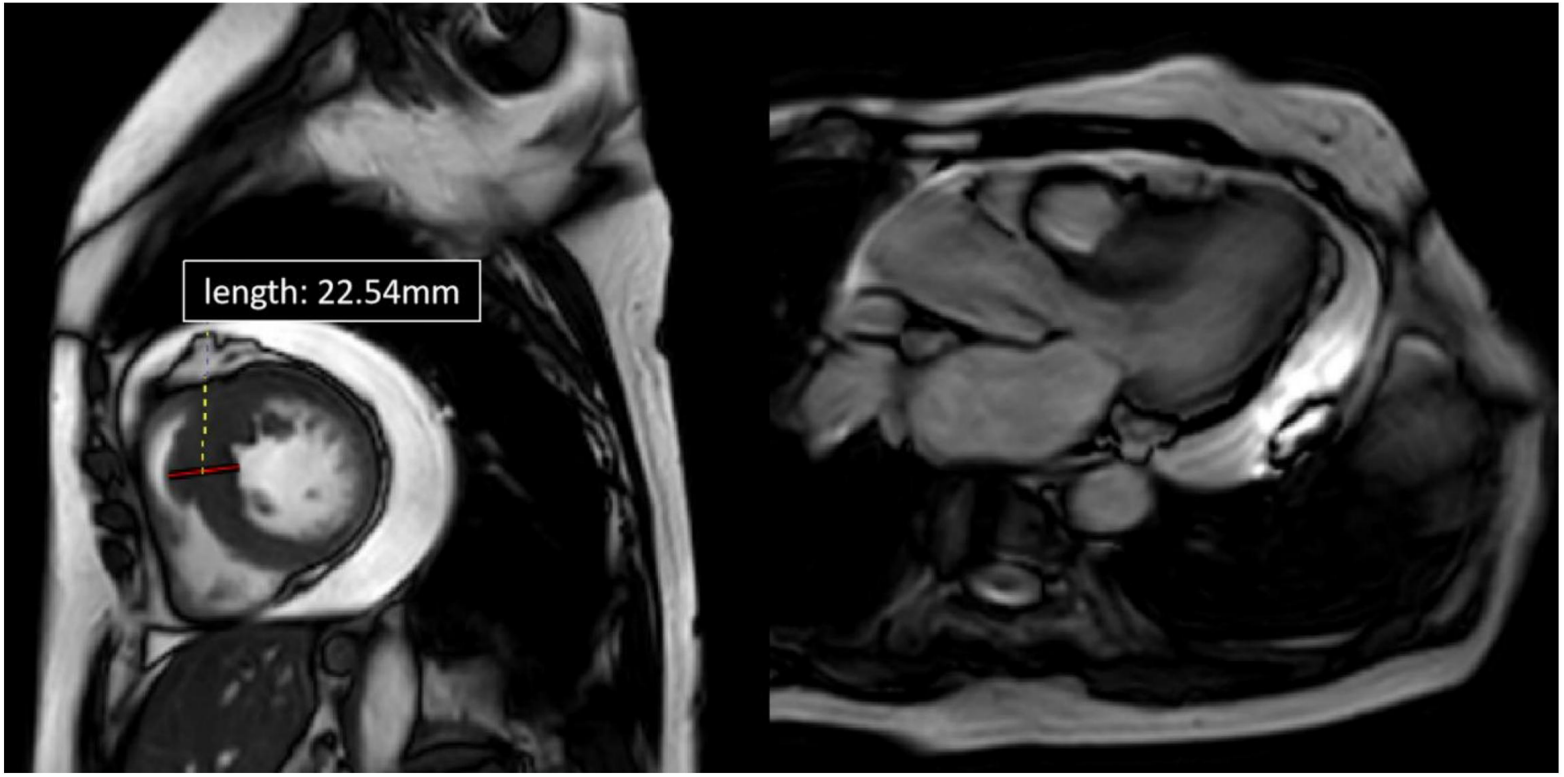
Cardiac MRI of patient 2, four-chamber view. Depicted long- and short-axis cine images. The thickest segment in the basal lower septum measuring approximately 22.54 mm. The left ventricular outflow tract (LVOT) was narrowed. Pericardial effusion was observed, with the deepest area measuring about 23.5 mm.

Upon admission, the patient was in a state of poor consciousness. A systolic ejection murmur was audible over the lower left sternal border, and there was evident edema in both lower limbs, though no signs of neck vein engorgement were observed. Her BNP level soared to 561.07 pg/ml. The dynamic electrocardiogram revealed severe sinus bradycardia, with an average heart rate of 43 bpm, a minimum heart rate of 32 bpm, and 1,793 episodes of sinus arrest (RR > 2 s). Both cardiac perfusion MRI (Figure 3) and echocardiography demonstrated hypertrophic cardiomyopathy and moderate pericardial effusion (Left ventricular posterior side: 16 mm, right atrial side: 13 mm). The thickest segment in the basal lower septum measured approximately 21.4 mm. After the Valsalva maneuver, the maximum flow velocity was around 201 cm/s, with a peak pressure gradient of approximately 16 mmHg.

We found no signs of tumor, inflammation, infection, or autoimmune diseases in this patient, leading us to hypothesize that the pericardial effusion was idiopathic. Idiopathic pericardial effusion in HCM patients is rare and may be associated with the impaired cardiac diastolic function and heart failure. To manage the complex comorbidities, a dual-chamber pacemaker was implanted on March 28, 2023 (the 11th day of hospitalization), with the consent of the patient and her family. By the fifth postoperative day, the patient's symptoms had significantly improved. The patient has been regularly followed up at our outpatient clinic, with echocardiograms showing reduced LVOT obstruction and no further increase in pericardial effusion. 21 days postoperative re-examination of echocardiography: resting LVOT forward flow velocity: 199 cm/s, no further increase after Valsalva; pericardial effusion: left ventricle posterior side: 14 mm, right ventricle anterior side: 4 mm. She said the chest pain decreased greatly and denied dizziness anymore.

## Discussion

Hypertrophic cardiomyopathy (HCM) is the most common inherited cardiomyopathy, primarily characterized by varying degrees of left ventricular outflow tract (LVOT) obstruction. The main symptoms of HCM include chest pain, dyspnea, syncope, and palpitations. HCM patients frequently exhibit various tachyarrhythmias (2), with atrial fibrillation and rapid ventricular arrhythmias being the most common causes of sudden cardiac death (SCD). Conversely, bradyarrhythmias such as sinus bradycardia, sinus arrest, and atrioventricular conduction block are considered uncommon in HCM patients. However, retrospective analyses and isolated cases have been reported. Zhang et al. (2) analyzed incidence rate and types of primary bradyarrhythmias in 101 HCM patients and found that 29 (28.7%) had primary bradyarrhythmias, including 17 (58.6%) with sinus node dysfunction (SND), 14 (48.3%) with atrioventricular block (AVB), and two (6.9%) with both SND and AVB. Additionally, 22 patients received pacemaker implantation—12 for AVB and 10 for SND—though none were for improving HCM-related symptoms. Furthermore, Barriales-Villa and colleagues (3) conducted a study to determine the prevalence of severe arrhythmias in 451 HCM patients, identifying 20 with SND and 28 with AVB.

Idiopathic pericardial effusion (i-PEF) in patients with HCM is relatively uncommon, and its underlying mechanism remains unclear. It may be related to the increase of pulmonary and systemic circulation pressure caused by cardiac diastolic dysfunction, and the increase of capillary plasma ultrafiltrate in pericardium and epicardium, which exceeds the corresponding lymphatic recycling limit. Besides, cytokines and inflammatory cytokines may also involved in pericardial effusion in patients with poor cardiac function (4). Dr. Puwanant (5) was the first to explore the prevalence of i-PEF in HCM patients, as well as the clinical and pericardial pathological characteristics of these individuals. Their research revealed a prevalence of moderate to severe pericardial effusion in 4% of HCM patients. Compared to HCM patients with no or minimal pericardial effusion, those with moderate to large i-PEF were younger and more likely to exhibit right ventricular hypertrophy, a greater maximal septal thickness, and pulmonary hypertension.

Most HCM patients’ symptoms can be mitigated with medication, usually involving beta-blockers or calcium channel blockers as the first-line therapy, with disopyramide acting as a second-line treatment. In recent years, myosin inhibitors, such as Mavacamten, have been developed to treat HCM by reducing LVOT obstruction and enhancing diastolic function (6, 7). When pharmacological therapy proves ineffective, invasive interventions may be considered. These invasive treatments can be categorized into procedures that modify the septal structure, such as septal myectomy (SME), alcohol septal ablation (ASA), and septal radiofrequency ablation, as well as dual-chamber pacemaker implantation, which alleviates LVOT obstruction by altering the sequence of myocardial contraction. Each technique possesses distinct advantages and indications. SME remains the gold standard for HCM treatment, effectively reducing LVOT obstruction by resecting a portion of the septum. ASA entails injecting anhydrous alcohol into the septal perforator artery that supplies the hypertrophied septum, leading to selective occlusion and subsequent ischemic necrosis of the hypertrophied septal myocardium, thereby diminishing myocardial contractility. Some studies indicate that ASA may not be as effective as SME in reducing LVOT obstruction and improving cardiac function. Additionally, ASA carries the risk of causing AVB (8) and may necessitate reoperation (9). Furthermore, patients lacking septal perforating branches are ineligible for alcohol ablation (10). Septal radiofrequency ablation can be executed using various methods, including catheter-guided or ultrasound-guided percutaneous approaches, to induce localized vascular and myocardial necrosis, thereby alleviating obstruction (11, 12).

Dual-chamber pacing therapy has been utilized in clinical practice for an extended period, with numerous studies and clinical cases demonstrating its efficacy in improving obstruction, reducing the LVOT pressure gradient and peak flow velocity, preserving cardiac function, and downgrading New York Heart Association (NYHA) class in patients with HCM (13, 14). The mechanism of LVOT obstruction in HCM patients primarily involves mechanical obstruction due to septal hypertrophy and dynamic obstruction resulting from systolic anterior motion (SAM) of the mitral valve. The right ventricular apex of dual-chamber pacing initiates earlier contraction of the right ventricle, from which the electrical signal spreads sequentially through the septum and onto the left ventricle. By the time the excitation reaches the left ventricle, the septum has already completed its contraction and no longer protrudes into the LVOT, thereby enlarging the outflow tract and alleviating systolic LVOT obstruction. This reduction in the pressure gradient also diminishes the SAM of the mitral valve leaflet during systole, further reducing outflow tract obstruction and increasing cardiac output, thereby relieving the patient's clinical symptoms. While alleviating obstruction, the electrode simultaneously located in the right atrium ensures atrioventricular sequential contraction through atrioventricular delay, improving diastolic function (15) and increasing coronary artery perfusion and cardiac output.

In our two patients, the pressing issues are sinus arrest and LVOT obstruction. Due to the presence of bradycardia and sinus arrest, first-line treatment drugs such as beta-blockers or calcium channel blockers are relative contraindicated. While mavacamten can increase LVOT obstruction and heart function, for these patients, it does not address the issue of bradycardia, and there is no evidence to indicate that it is safe and effective for patients with bradycardia. Among the invasive treatment methods for hypertrophic cardiomyopathy, pacemaker has the least surgical trauma and anesthesia risks, and the risk of postoperative arrhythmias is also low. Additionally, the approach for treating bradycardia in patients is undoubtedly the pacemaker. Since the patients’ sinus node dysfunction, a dual-chamber or triple-chamber pacemaker (i.e., Cardiac Resynchronization Therapy-Pacemaker, CRT-P) is more suitable. In the meanwhile, three-chamber pacemaker can alleviate LVOT obstruction and reduce ventricular remodeling (16) by improving cardiac diastolic function, especially suitable for patients with left bundle branch block (LBBB) and reduced ejection fraction (17, 18). Preliminary observational clinical studies have shown that conduction system pacing, such as His bundle pacing (HBP) and left bundle branch pacing (LBBP) (19), which have been gradually developed in recent years, can achieve similar efficacy to CRT in terms of providing electrical synchrony, improving left ventricular ejection fraction, and correcting heart failure (20, 21). And the cost is lower, which is more consistent with physiological pacing. However, CRT, HBP and LBBP are all biventricular synchronous pacing, which cannot create the paradoxical motion of artificial ventricular septal and left ventricular wall that is caused by right ventricular pacing, nor can it alleviate the LVOT obstruction that results from it.

Regarding the implantation of a dual-chamber pacemaker, we have also considered its drawbacks. One of them is that long-term high rate right ventricular pacing and the resultant asynchronous ventricular contraction will increase the risk of heart failure (22), arrhythmia, and pacemaker-induced cardiomyopathy (23). In addition, there have studies to prove that Dual-chamber pacing offered less reduction in LVOT gradients and improvements of patient symptoms and functional status when compared with myectomy (24) and ASA (25). Additionally, there have studies even suggested that the benefits of pacemaker therapy might be attributable to a placebo effect (26, 27).

Given that our two HCM patients are elderly (>65 years old) and have sinus node dysfunction. Sinus arrest is the greatest risk of sudden cardiac death for them. Although pacemaker therapy may have a limited effect on reducing obstruction, it can correct the heart rate. With reference to the 2024 AHA guidelines for hypertrophic cardiomyopathy and 2021 ESC Guidelines on cardiac pacing (17, 18), and with the consent of the patients and their families, we have performed dual-chamber pacemaker implantation for these two patients. Fortunately, our patients’ treatment outcomes are encouraging. In our cases, the pacemaker served three critical functions: (1) effectively addressing the patient's bradycardia and sinus arrest, thereby preventing SCD certainly and ensuring the continued safe use of negative inotropic drugs; (2) reducing LVOT obstruction through changing the order of ventricular contraction; (3) improve diastolic function and reduce pericardial effusion. Therefore, for HCM patients who are refractory to or have contraindications for medication, the optimal invasive treatment approach is not one-size-fits-all. Physicians must conduct thorough examinations and comprehensive evaluations to tailor the most appropriate treatment for each individual, taking into account factors such as the extent and location of septal hypertrophy, coronary septal branch anatomy, valve morphology, age, comorbidities, surgical risks, and patient preferences. For patients with arrhythmias, the risk of SCD should be fully assessed. Pacemaker therapy is undoubtedly beneficial for HCM patients with sinus node dysfunction, and it can also be advantageous for those with idiopathic pericardial effusion.

## Conclusion

We presented two elderly patients with hypertrophic cardiomyopathy (HCM) accompanied by bradyarrhythmia, one of whom also exhibited moderate pericardial effusion. Following dual-chamber pacemaker implantation, both patients demonstrated significant improvement in symptoms and clinical signs. Echocardiography revealed a reduced left ventricular outflow tract (LVOT) gradient. Additionally, in the patient with idiopathic pericardial effusion, there was no significant progression of pericardial effusion, which may be linked to the alleviation of obstructive symptoms and the prevention of further exacerbation of severe bradyarrhythmia and heart failure. To our knowledge, we are among the few to report on HCM associated with bradyarrhythmia. Although the indications for dual-chamber pacing in the treatment of HCM are significantly limited, we believe this therapeutic approach remains essential and can be considered a first-line treatment for the subgroup of elderly patients with LVOT obstruction accompanied by bradyarrhythmia.

## Data Availability

The raw data supporting the conclusions of this article will be made available by the authors, without undue reservation.
