# Supplementary material for: Case Report: Dual-chamber pacemaker for hypertrophic cardiomyopathy with bradyarrhythmia and idiopathic pericardial effusion: a report of two cases and literature review
Source: Front Cardiovasc Med. 2025 Feb 27;12:1518000. doi: 10.3389/fcvm.2025.1518000 (PMC11903486; doi:10.3389/fcvm.2025.1518000)
Supplement: Supplementary file 1 [file Datasheet1.pdf]

Timeline

	Patient 1	Patient 2
12 years before dmission		Diagnosed with hypertrophic cardiomyopathy
4 years before dmission		Diagnosed with sinus bradycardia
Upon admission	Diagnosed with hypertrophic cardiomyopathy with sinus bradycardia firstly: a echocardiography revealed the maximum LVOT pressure gradient of 111 mmHg, maximum flow velocity of 528 cm/s; the Holter detected bradycardia accounting for approximately 17.5% of the total heartbeats, and four sinus arrests	Cardiac MRI and echocardiography demonstrated the maximum flow velocity of LVOT was around 201 cm/s, with a peak pressure gradient of approximately 16 mmHg, and moderate pericardial effusion (Left ventricular posterior side: 16mm, right atrial side: 13mm); a electrocardiogram revealed severe sinus bradycardia, with 1793 episodes of sinus arrest
Day 11 in the wards		Dual-Chamber pacemaker implantation
Day 13 in the wards	Dual-Chamber pacemaker implantation	
Day 3 after pacing	The greatly decreased maximum LVOT pressure gradient of 24 mmHg; the holter indicated pacemaker working well, pacing heart rate accounting for approximately 17.5% of the total heartbeats, with an average heart rate of 67 bpm, a minimum heart rate of 59 bpm, and no instances of sinus pause.	
Day 4 after pacing		Discharge.
Day 8 after pacing	Discharge	
Day 21 after pacing		Resting LVOT forward flow velocity: 199cm/s, no further increase after Valsalva; pericardial effusion: left ventricle posterior side: 14mm, right ventricle anterior side: 4mm.
Day 75 after pacing	BNP：81.11pg/ml.UCG：resting LVOT forward flow velocity: 244 cm/s.	
Day 80 after pacing		Resting LVOT forward flow velocity: 158cm/s, no further increase after Valsalva; pericardial effusion: Left ventricular posterior side: 17mm, right atrial side: 11mm
Day 122 after pacing		Resting LVOT forward flow velocity: 177cm/s, no further increase after Valsalva; pericardial effusion: Left ventricular posterior side: 15mm, right atrial side: 9mm
